# Exploring HIV concern in a population of Dominican American women midlife and older

**DOI:** 10.1186/s12889-019-7810-1

**Published:** 2019-10-31

**Authors:** Michelle Odlum, Danielle Black, Sunmoo Yoon, Cassidy Maher, Steven Lawrence, Jennel Osborne

**Affiliations:** 1 Columbia University School of Nursing, 560 W 168 St, New York, NY 10032 USA; 2 Columbia University Medical Center, New York, NY 10032 USA; 3 Columbia University Mailman School of Public Health, 722 W 168th Street, New York, NY 10032 USA

**Keywords:** Latino women, HIV risk, Aging, gender and power, Dominican Republic

## Abstract

**Background:**

The feminization and ethnic diversification of HIV infection, has resulted in a call for gender- and culture-specific prevention strategies for at-risk groups including Latinos in the United States. The steadily changing demographic profile of the AIDS epidemic challenges prevention strategies to remain relevant and up-to-date, particularly in populations of women midlife and older where an understanding of risk remains under explored. As the CDC requests country-specific HIV risk profiles for Latino communities in the US, understanding the socio-economic, behavioral and personal risk reasons of HIV risk for older Dominican women is critical for prevention.

**Methods:**

We conducted focus group discussions informed by the Theory of Gender and Power (TGP). The three constructs of the TGP: 1) Affective influences/social norms; 2) Gender-specific norms and.

3) Power and Authority guided the thematic analysis and identified themes that described the socio-cultural and contextual reasons that that contribute to perceptions of HIV risk.

**Results:**

Sixty Dominican American women ages 57–73 participated in our focus group discussions. Sexual Division of Labour: 1) Economic Dependence; 2) Financial Need and 3) Education and Empowerment. Sexual Division of Power: 4) HIV Risk and 5) Relationship Dynamics. Cathexis: Affective Influences/Social Norms: 6) HIV/AIDS Knowledge and 7) Prevention and Testing. Importantly, participants were concerned about partner fidelity when visiting the Dominican Republic, as the country accounts for the second highest HIV rates in the Caribbean.

**Conclusions:**

Our results confirm previous findings about perceptions of HIV risk and provide additional insight into aging-related aspects of HIV risk for Latino women midlife and older.

## Background

In the United States (US), Latino communities bear a disproportionate share of the HIV burden. Latinos comprise 17% of the US population, but account for 23% of the people living with HIV and AIDS (PLWH), with an estimated 21% of all new infections annually [1, 2]. If rates of infection persist, one in 50 Latinos will be diagnosed with HIV during their lifetime. Although men account for 87% of new HIV infections in US Latino communities, disturbing trends emerge among women [1, 2]. HIV rates among Latino women are four times that of White women, with heterosexual intercourse being the primary mode of transmission [1, 2]. Cultural values promoting inequitable gender norms, along with other risk reasons, create a unique configuration for HIV risk among Latino women [3, 4]. Furthermore, additional attention must be given to women midlife and older, where significant increases in the rates of HIV acquisition have been observed. Studies on HIV and women indicate that rates are declining for almost all women except those over 50 [5–7]. For Latino women over 50 years if age, increased risk is associated with aspects of aging and culture [3]. Age presents additional population-specific risk reasons contributing to poor utilization of diagnostic, treatment and prevention services [8, 9]. With over half of the 1.1 million PLWH in the US age 50 years of age and over, and rates for older Latinos five times higher than their non-Hispanic White counterparts, sexual risk behaviors of older Latino women must be explored to inform targeted prevention efforts [3, 8].

The full determinants of HIV risk in US women are not completely clear, particularly in populations of women midlife and older [5]. Studies that attempt to use theoretical frameworks for behavioral predicators of HIV risk in women have focused on the younger women of adolescence and reproductive age [10, 11]. Yet, considerable measurement errors exists to capture demographic characteristics, beliefs and attitudes in studies of younger women, where a variety of other reasons contribute to risk behavior (e.g., pregnancy) [9, 11]. This results in the inability to quantify and relate observed effects to women midlife and older [5]. Intervention efforts and prevention programs have traditionally excluded older age groups as well. Many women midlife and older are sexually active [5]. In past populations of older adults, sexual activity was not discussed or deemed inappropriate [5]. However, the sexual revolution of the 60s and 70s, led to different beliefs regarding sexual expression among baby boomers [12]. These and other steadily changing reasons associated in the demographic profile of the AIDS epidemic, challenges HIV prevention strategies to remain relevant and up-to-date [13]. Moreover, the ethnic diversification of HIV in US women, have resulted in a call for the development and evaluation of gender- and culture-specific HIV prevention strategies [13, 14]. Research in populations of Latino women exclude an understanding of the psychosocial reasons related to older women and HIV [1, 2]. In fact, older Latino women are one of the least studied American demographic groups with regard to social, health or sexual behavior [1, 2, 15]. Knowledge gaps can leave health professionals ill-prepared to effectively discuss HIV/AIDS prevention [3, 4, 16].

We sought to identify perceptions of HIV risk in a US-based immigrant population of Dominican women age 50 and older living in the Northeast, where the HIV diagnostic rates in women remain high [1]. The vulnerability to HIV in older immigrant Dominican women remains under explored. Guided by the Theory of Gender and Power, our study sought to explore the perceptions of HIV risk, and to identify themes that describe the socio-cultural and contextual reasons that contribute to perceptions of risk. Theoretical construct analysis that leads to important mediators of behavior change can be viewed in the context of personal characteristics, such as age, and can assist in the intervention development and refinement processes. Our study is timely as the Centers for Disease Control and Prevention (CDC) recently called for an understanding of country-specific perceptions of HIV risk among Latinos, as such perceptions may lead to different risk profiles and preventive behaviors [1, 17].

The current study was a sub-study of the Washington Heights/Inwood Informatics Infrastructure for Community-Centered Comparative Effectiveness Research (WICER) [18, 19]. The WICER study included a longitudinal patient household health survey, administered to over 5000 Washington Heights/Inwood (WH/I) community residents of New York City [18, 19]. To encourage ongoing involvement, participants were asked to list their three top health concerns. Over 921 female participants listed HIV as a top health concern ranking it fourth overall behind diabetes, hypertension and heart disease. Survey demographics indicate these respondents were mainly female immigrants of Dominican descent ages 50 and over [18, 19]. To identify themes that describe the socio-cultural and contextual reasons that contribute to perceptions of risk, we recruited WICER women ages 50 and over to participate in focus group discussions.

### Theoretical framework

The Theory of Gender and Power (TGP) guided our qualitative research design and thematic analysis [20, 21]. The TGP focuses primarily on gender-based imbalances. It provides a framework to understand the socioeconomic, interpersonal, and individual exposures that place women at increased risk for sexually transmitted infection (STI) acquisition, including HIV. The theory provides insight into the manifestation of gender-based structural inequalities in relationships. In the context of HIV risk, the theory suggests that gender-based power imbalances are pervasive in society and several social-structural and cultural reasons empower men, giving them interpersonal decision-making control in social circumstances including intimate relationships. According to the TGP, three distinct concepts impact women’s HIV risk. These are Gender Specific Norms [20, 21] (Labor), Power & Authority and Cathexis (social norms and affective attachments). Gender Specific Norms (Labor) refers to inequalities in the control of resources by women, such as financial and employment related reasons [20, 21]. Power & Authority refers to inequalities in power and control that exist between men and women in society that favor men. These include feelings of powerlessness in relationships or in one’s perceptions of their ability to thrive [20, 22]. Cathexis: Affective Influences/Social Norms refers to cultural normative roles and social influences that may further weaken a woman’s role and increase inequality [20, 22]. We utilized the TGP defined concepts that impact women’s HIV risk to inform our data collection, analysis, and interpretation of findings.

## Methods

### Study design and data collection

A convenience sample of WICER women 50 years of age and over (≥50) was recruited to participate in the HIV concern study activities. HIV risk surveys were mailed to the homes of WICER women ≥50 who identified HV as a top health concern (*N* = 700). Survey completers (*N* = 350) were invited to participate in focus group discussions. Qualitative data was collected in the form of 6 focus groups with 60 participants. Focus group discussions of 10 adults is considered ideal. Groups that are too large may be difficult to control and to achieve meaningful interactions. Smaller groups may reduce the opportunity for varied inputs. Therefore, six focus groups totaling 60 adults: 1) ensures variability on potentially important characteristics, 2) achieves adequate theoretical saturation, and 3) permits identification of important theoretical propositions related to the study’s objectives. WICER women ≥50 were targeted due to their identified concern of acquiring HIV from the original WICER household survey. Demographic data, collected on the original WICER household survey was updated at the focus group sessions. Inclusion criteria for focus group participation also included literacy in English and/or Spanish and the ability to provide informed consent. The final study protocol was reviewed and approved by the Institutional Review Board at the Columbia University Medical Center. Focus groups were facilitated by two bilingual Research Assistants. Written informed consent was obtained from all participants included in the study. Participants were given the option to join focus group discussions conducted in either English or Spanish, based on participant preference. Groups lasted approximately 60–90 min and participants were provided food and an incentive (fifty-dollar grocery coupon) for their time.

### Instrument

Our focus group guide followed a structured protocol of eight open-ended questions, informed by the Theory of Gender and Power. Questions were designed to elicit the perspective of participants on reasons related to HIV risk in the Dominican community. Focus group questions, with corresponding probes, assessed HIV/AIDS knowledge, attitudes and beliefs (e.g., When you think about HIV & AIDS, what comes to your mind?), cultural norms (e.g., Tell me some other reasons/things in Dominican culture that influences the spread of HIV/AIDS in women.) and gender norms (e.g., What are some reasons related to aging that can cause difficulties in intimate relationships?).

### Statistical analyses

Descriptive statistics were used to describe the demographics of the focus group participants. Focus group audiotapes were transcribed. Spanish focus groups were transcribed verbatim in Spanish then translated into English. Three analyses were conducted on focus group data in order to identify similar, separate and distinct findings, to inform a thorough understanding of population-specific HIV concern. Our unit of analysis were focus group participants. We utilized data science methods (content analysis and sentiment analysis) and traditional methods (thematic analysis). Coding and thematic analysis of the transcripts was supported by NVivo 8.0 software and the R software program. Content analysis and sentiment analysis were conducted, utilizing R software, on all transcripts by applying natural language processing, to determine frequently discussed words and to detect potential themes. Focus group transcripts were filtered to remove stop words (e.g., at, does, for) and punctuation, to identify topics and transformed text to a vector form and N-gram, followed by reducing the dimensionality of the volume. Data cleaning included the removal of nonstandard or special characters that may hinder the use of content mining software. N-gram, an example of features, is a subsequence of N items in a given sequence from characters to words. A term-frequency dictionary was computed with the N-gram method. Preprocessed data was then analyzed. The most frequent keywords were identified with frequency counts, this process is not focus group participant specific. The N-gram forms (unigram, bigram and trigram) of keywords that commonly occurred together were clustered based on content similarities for topic detection. Frequent keywords relevant to the TGP constructs were identified by our team’s Data Scientist (SY) and confirmed by the topic expert (MO, DB). The sentiment of frequent keywords was then analyzed. To assess sentiment in high frequency words were aggregated into categories: 1) Supportive (e.g., praising); 2) Negative (e.g., expressing distaste) positive and negative scores were then generated. Based on our categories, our sentiment analysis will generate human emotional valance scores falling on a range from − 10 to + 10. For example, highest scores + 10 for the most Supportive and lowest scores − 10 for the most Negative and 0 for Unrelated. The positive and negative scores for all focus group transcripts combined. Clusters were visualized to summarize the detected topics using infographics.

Thematic analysis was also conducted, utilizing NVivo, to identify recurring patterns within the data, based upon a priori themes related to the Theory of Gender and Power and additional themes that emerged during the coding process. The coding framework was used to interpret and explore the data from all ten transcripts and to identify experiences and perceptions of HIV/AIDS that would affect risk. Coding schemes were reviewed and discussed with team members with expertise in HIV/AIDS, thus shaping the final coding structure. Synthesis of data occurred across focus groups. Cohen’s Kappa, a measure of the agreement between two raters, was then determine by two additional team members. Themes were then organized under the TGP constructs of Affective Attachment, Division of Power and Gender Specific Norms (Labor).

## Results

### Characteristics of the study population (WICER survey items)

Our sample included 60 women ages 50 and over, with a mean age of 64.0 years (SD ± 12.0, range = 50–73 years). Most were born in the Dominican Republic (86.9%) and reported their primary language as Spanish (100%). Relationship status of participants were reported as Married (*n* = 19, 31.3%), Living with Partner (*n* = 4, 5.3%), Single (*n* = 22, 37%), Divorced/Separated (*n* = 1, 1.4%) and Widowed (*n* = 3, 4.9%), (20.1%); with an average of 4 living in each household (SD = 3.6; range = 1–16). Most participants identified their education level as some high school or less (*n* = 26, 43.1%) high school diploma/GED/Some College (*n* = 26, 43.5%) and College graduates (*n* = 8, 13.4%). Only (*n* = 19, 31.7%) had adequate health literacy (Newest Vital Signs- validated health literacy measure). Less than half of the participants (*n* = 27, 44.4%) worked outside the home. Of those that did not work outside the home (*n* = 8, 13.3%) identified themselves as homemakers looking for work, (*n* = 9, 14.1%) as retired, (*n* = 4, 6.9%) as students and (*n* = 4, 6.9%) as disabled (*n* = 4; 6.9%).

### Sentiment and content analysis

**Fig. 1:**
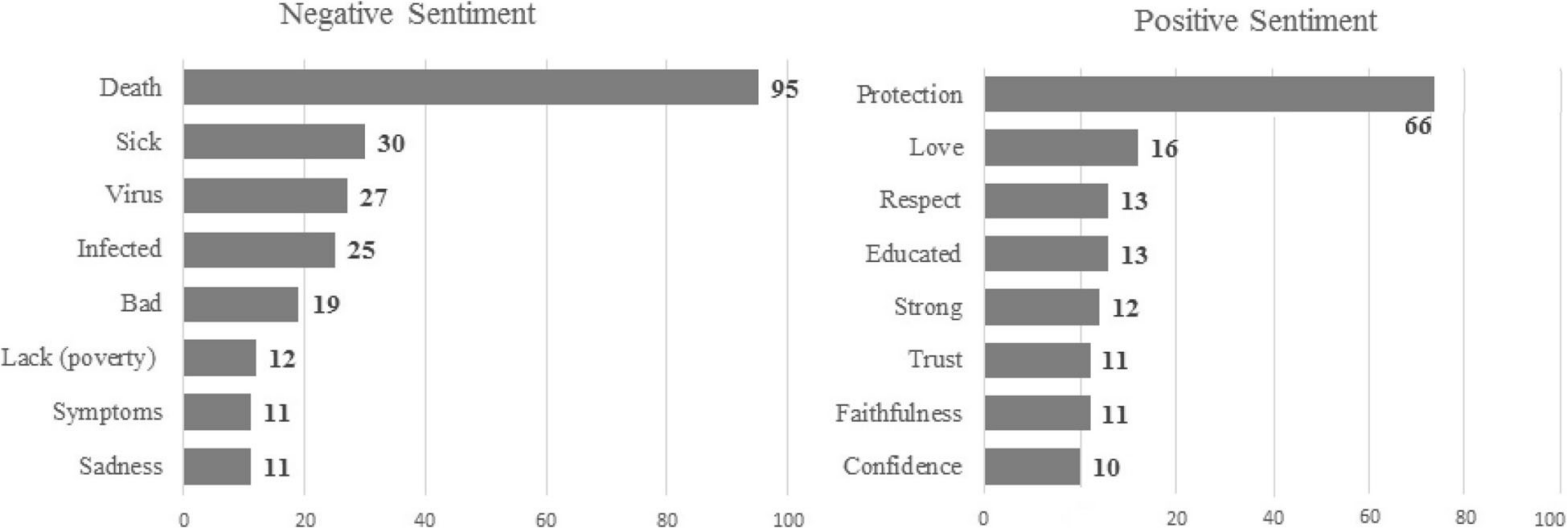
Negative and positive sentiment from focus group discussions

**Fig. 2:**
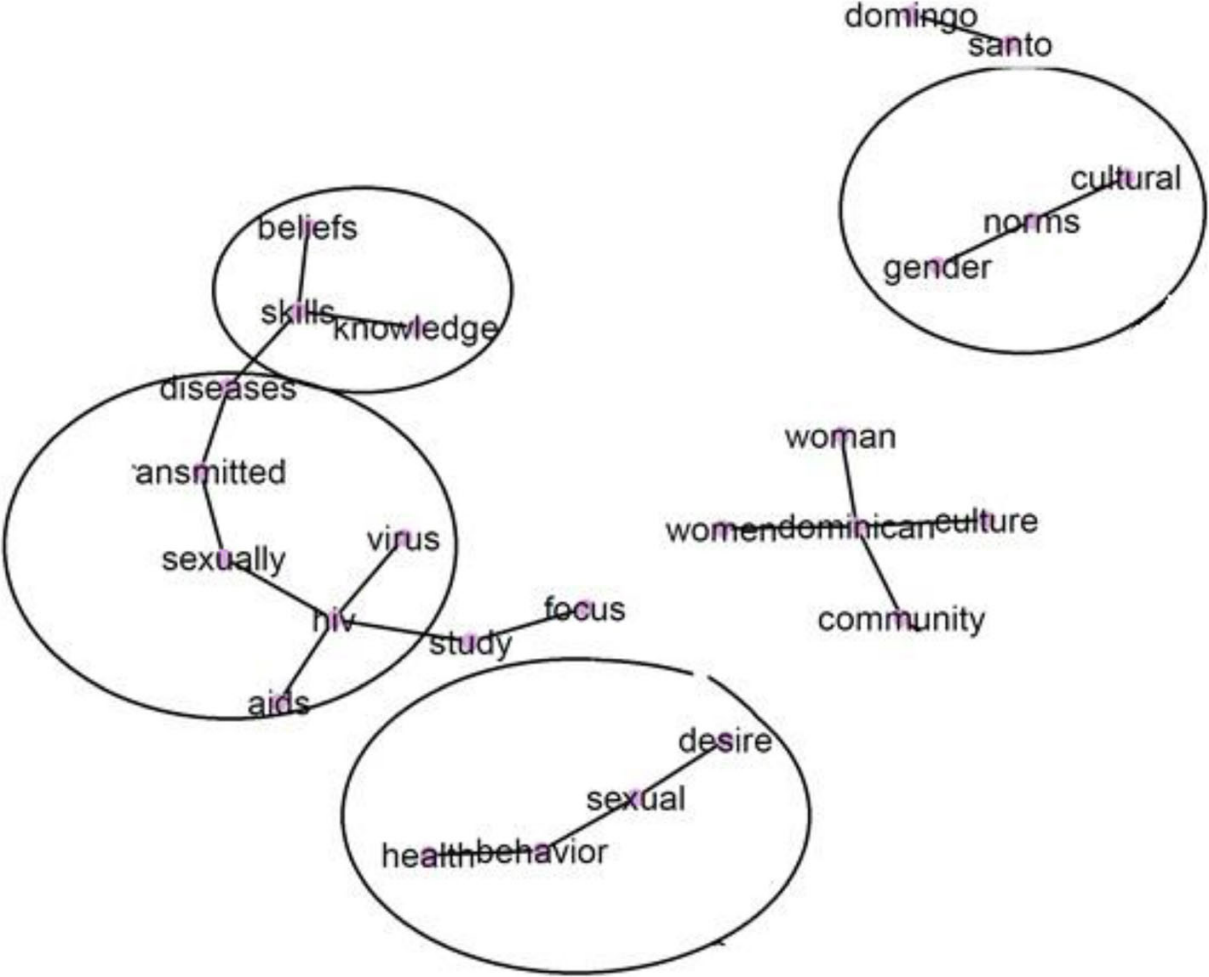
Most frequent word clusters from focus group discussions

Our sentiment analysis, identified HIV-related sickness and death as the top negative sentiment. Directly related to the stories told by participants of adults they knew who contracted the virus in the US and the Dominican Republic. They also indicated that poverty increased HIV infection. The greatest positive sentiment was protection, which frequented discussions around partner fidelity needing to be maintained while visiting the Dominican Republic. Participants also identified protective reasons that included aspects of relationship health (e.g., respect and faithfulness), in addition to women having strength and education, Fig. 1. Content analysis results revealed the most frequent word clusters, connected by lines and circled for easy viewing and understanding, of our focus group discussions. Participants spoke most about: 1) the cultural and gender norms in their community; 2) sexual and health behaviors and desires; 3) AIDS and other sexually transmitted diseases; and 4) knowledge beliefs and self-efficacy skills, Fig. 2.

### Thematic analysis

**Fig. 3:**
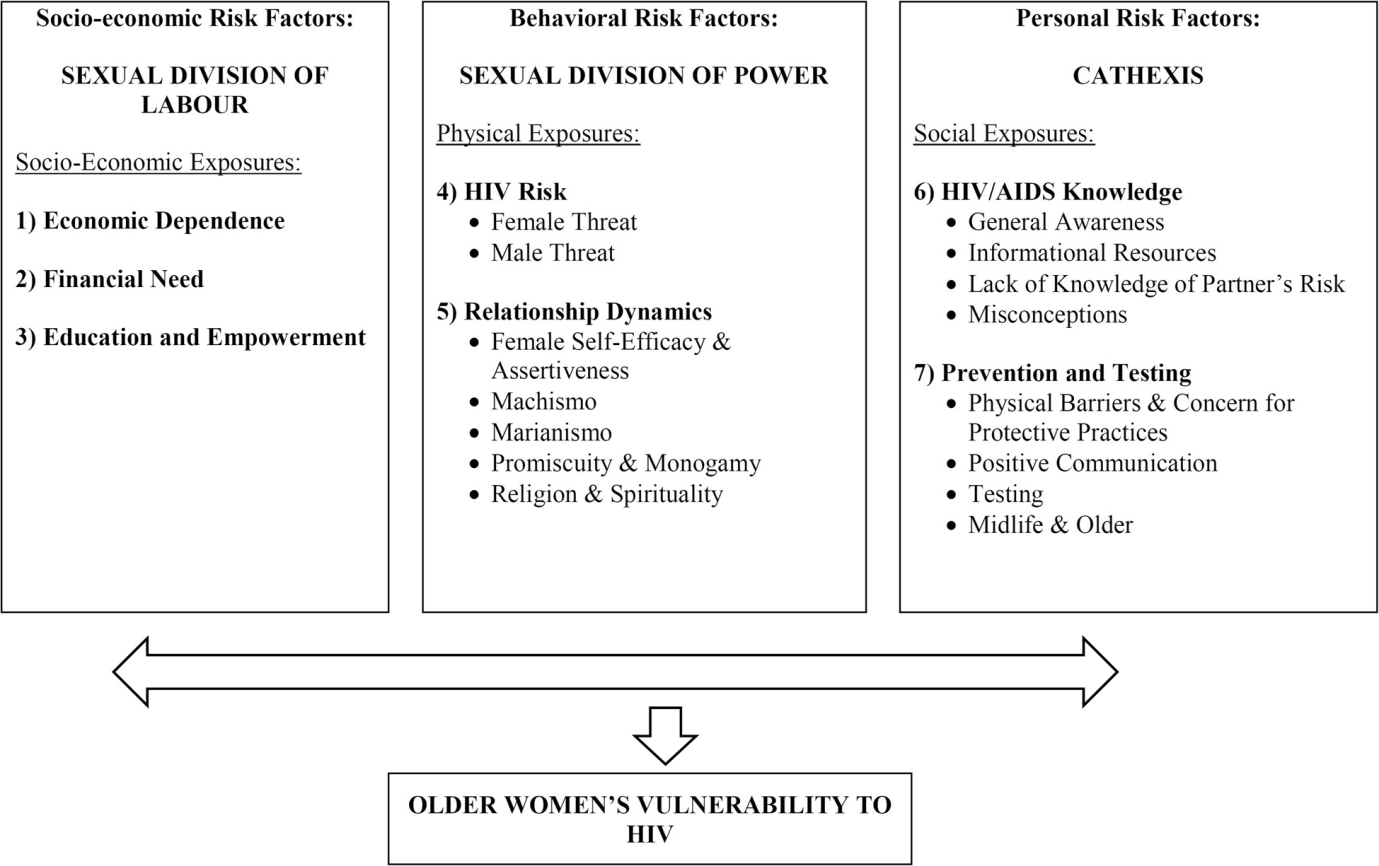
Theory of Gender and Power constructs with the identified themes from focus group discussions

Guided by the Theory of Gender and Power, eight overarching themes emerged. Cohen’s kappa was 0.62, indicating a substantial level of agreement. Differences may reflect the academic disciplines of the raters Statistical Methods (KM) and Biological Sciences (SL)). The overarching themes found were: **Gender Specific Norms (Labor):** 1) Economic Dependence; 2) Financial Need; 3) Education and Empowerment. **Power & Authority:** 4) HIV Risk and 5) Relationship Dynamics. **Cathexis: Affective Influences/Social Norms:** 6) HIV & AIDS Knowledge; and 7) Prevention and Testing, with the horizontal arrow indicating bi-directionality of the TGP constructs and the vertical arrow indicating the direct relationship of the TGP constructs to HIV vulnerability Fig. 3.

### Gender specific norms (labor)

There are a variety of economic-related situations that were believed to increase older Dominican women vulnerable to HIV risk. ***Economic Dependence*** Participants discussed their dependency on their partners for their livelihood and the importance their sexual relationship plays in their finances. One participant stated; “He had to leave me [money] every day on the table first, if not, I did not sleep with him. ***Financial Need*** was also identified as a critical necessity that directly impacts their vulnerability to their partners, therefore resulting in an increase in sexual risk. One participant eloquently stated, “And they are things, you know, that - and there are many, many things that Dominican women do sometimes out of necessity, sometimes by choice, sometimes because they are women of the street.” Educational barriers to employment and sustainment of livelihood were also identified as an economic-related reason that contribute to risk. Participants discussed their inability to read and write in English and the need for language classes for financial stability. However, participants also understood that ***Empowerment through Education*** is the optimal way of protection from HIV/AIDS, with a participant stating concisely, “It [risk] depends on the degree of education of women.”

### Power & Authority

Participants highlighted their understanding of HIV risk in the Dominican community. The identified reasons speak directly to relationship dynamics of older Dominican women. They included: 1) Female Threat, and 2) Male Threat. ***Female Threat*** was identified as risk of HIV transmission attributed to relationship seeking based on a variety of need-based reasons*.* A participant concisely stated, “There are women who are seriously looking for men, even if they are married, they don’t care. The men then get infected with the virus and then transmit AIDS to the wife at home.” This was followed by statements of ***Male Threat*** connected to promiscuity and infidelity. A participate highlighted, “When a man feels that he can sleep with any woman because he does not care. He can sleep with many women and not care if he is infected or if he is infecting other women, because he is a man and that is what men are supposed to do.” Others identified transmission risk to be associated with an ongoing link to the Dominican Republic. With the HIV/AIDS epidemic on the rise in the Dominican Republic, the transient nature of the community with continual visitations home, place women at higher risk due to Male Threat [https://www.aidshealth.org/global/dominican-republic/]. A participant explained, “Yes, for example, when they go to Santo Domingo those men go crazy.”

“The wife is powerless, because sometimes the husband will go to Santo Domingo and having his wife here, they go there looking for a girl. They send your money to these women”.

Participants explained the important role of Relationship dynamics in the Dominican community: 1) Female Self-Efficacy & Assertiveness, 2) Machismo, 3) Marianismo, 4) Promiscuity & Monogamy and 5) Religion & Spirituality. Participants discussed a variety of relationship dynamics that occur among older Dominican women and areas for improvement to reduce HIV risk among their peers. Participants mentioned the importance of issues regarding ***Female Self-Efficacy & Assertivene*ss** for HIV risk reduction, “If I say that we use condoms, we use condoms, or else nothing.” Although many stated the empowering nature of insisting on condom use in all focus group discussions, the barriers to this remained evident in their discussions. A variety of other themes emerged regarding increased risk of HIV, as the necessary assertiveness among older Dominican women is directly attributed to additional Power & Authority related community-level reasons that emerge in these subsequent themes. These include ***Machismo***, the encouragement of relationship dominance: “There are some marriages that the man - Is the boss.” ***Promiscuity*,** “Yes. You must be careful and know for sure that her husband is not someone who is looking (having sex) with other women.” ***Marianismo***, the encouragement of sexual passivity in women: “Women must adjust to what they say [men]. That happens. That is the reality of the Dominican man, who believe that women must be subjected to everything they say and do.” ***Monogamy****,* “Oh, and that women should have a single partner” and surprisingly, ***Religion & Spirituality***, “But you do not know. I like to think we’re good (my husband and I) I was sure that he was not seeing other women, because I am Christian and that is my belief. You just don’t know for sure.” These identified beliefs can impact the Power & Authority dynamics that may also increase risk in older Dominican women.

### Cathexis: affective influences/social norms

Participants believed HIV/AIDS Knowledge in the Dominican community of older women centers around: 1) General Awareness, 2) Informational Resources, 3) Lack of Knowledge of Partner’s Risk, and 4) Misconceptions. Participants identified individual and societal influences that impact HIV risk in older Dominican women and identified areas for improvement. A participant simply stated that ***General Awareness*** of HIV/AIDS Knowledge should center around protection, “If you know that you should not have sex without protection, then why have it? They have to be aware.” ***Informational Resources*** was also mentioned as critical to HIV Knowledge in the Dominican Community. An evaluation of HIV-related resources occurred across focus groups, with participants recognizing the source of HIV risk information. Media and technology were also mentioned, with a participant concisely stating “Agreed. … get tested, and watch television that provides much information.” Participants also believe ***Lack of Knowledge of Partner’s Risk*** and blatant denial contributed to increased risk. This is accepted behavior in their community. A participant provided a scenario of a Dominican widow, “He died, she did not know of what. They either lived together or were separated. She got sick, and did so many tests. Testing here and there, and they could not find out what was wrong. A doctor suggested, “We will give you the AIDS test,“ She asked, “Why?“ Because she was very offended.” Participants also identified other knowledge deficiencies including ***Misconceptions***, “Before I learned about the disease I thought that you can get it from a kiss or even touching hands …” Although participants identified sources of HIV information, the perpetuation of misconceptions among older Dominican women regarding transmission and even risk remain. This indicates a need for additional sources of HIV risk information and information tailored to include community-level reasons that are attributed to risk.

Participants explained aspects of prevention and testing associated with normative beliefs and affective influences in the Dominican community, responses centered on their challenges. Areas for improvement includes: 1) Physical Barriers & Concern for Protective Practices, 2) Positive Communication, 3) Testing and 4) Midlife & Older. Ways to prevent HIV as understood in the Dominican community were identified. ***Physical Barriers***, “It is very important to use condoms when you have a partner.” This was further reinforced by their ***Concerns for Protective Practices***, “It is a powerful disease, one has to protect themselves.” “And at this day and age, the man did not have to use protection because before relations were healthy, but not now.” When probed about other preventive strategies that occur within the community, ***Positive Communication*** between couples was also identified by many and viewed as a powerful preventive strategy, “Well, the couple should get to know each other well. The subject should be discussed; there should be a clear and open conversation. That is how you educate people in that regard.” ***Testing*** was also identified as important to HIV prevention. Participants recognized the barriers to protection that occur in perceived to be monogamous unions. Whether stated directly or indirectly, issues around fidelity, the need for HIV education and the change of normative beliefs to ensure women accept the importance of testing, was also recognized. This recognition occurred independent of relationship types. A participant eloquently stated, “What should be done first, even if you are getting married, know about what HIV/AIDS is and get tested too; because although they have a relationship, they have to have confidence to get tested.” Participants also felt the essential need for change in normative beliefs around aging. Although they identified effective ways to prevent HIV, some participants viewed women ***Midlife & Older*** to be less at risk for HIV infection. In fact, one participant stated, “I think, that we are from an era that we were not affected by it, thank the Lord.”

## Discussion

HIV infection risk is recognized to be on the rise in midlife and older women [6]. Culturally-specific HIV prevention programs are needed to address the unique context in populations of older women, where determinants of HIV risk are not completely clear [5, 23, 24]. The current study sought to use the Theory of Gender and Power to identify themes that describe the socio-cultural and contextual reasons that contribute to perceptions of HV risk in a population of Dominican women 50 years of age and older [20, 21]. Our study revealed a number of important findings that support the TGP constructs and inform population-specific interventions.

Our findings indicate a perception of economic dependence and financial need regarding partner dependence in our population of Dominican women. Identified in our study and based on the Gender Specific Norms construct, the ability to be financially independent allows for greater control and better negotiation in intimate partnerships. According to the literature, compared to other ethnic groups, Latino women in the US are more likely to be unemployed [4]. When employed they earn less and have limited opportunities for educational attainment, making them vulnerable to poverty and associated implications [3, 4, 25]. Studies report that higher levels of education results in greater financial stability and has protective effects on HIV risk [3, 7]. Moreover, adults with higher levels of education report a greater sense of control over their lives and health [3, 16, 26]. It is also found that women of color from low socioeconomic communities are at greater risk for HIV, with limited educational attainment directly associated with elevated risk [3, 4]. Language barriers further contribute to limited educational attainment in Latino populations and segregated neighborhoods [3, 16]. In fact, consistent condom use has been shown to be directly associated with both employment and higher education [3, 16]. Higher levels of education also increases the understanding of health information, and supports effective problem solving and improves coping skills [3, 26]. For women in unequal gender-based power relationships with dominant partners and heightened pressures, [3, 4] *as expressed by our study sample, recommendations that interventions focus on communication skill building and risk reduction negotiations may improve HIV-related outcomes.* To improve the comprehension of actual HIV risk, interventions should also provide an in-depth understanding of gender roles, generational reasons, and societal influences that contribute to relationship-specific vulnerabilities [12].

For **sexual division of power**, our participants discussed high risk sexual behaviors of both women and men. Participants indicated that women are motivated by a variety of circumstances which may result in unsafe sexual practices with multiple partners. A study in a population of Black and Latino women, who reported having multiple sexual partners, the older women were less likely to use condoms with their primary partners [4, 27]. Similar to our findings of high risk behavioral practices of both men and women, this study also suggests that multiple partnerships in older women may be at increased risk given primary partners’ behaviors [4, 16, 27]. The literature suggests a consistent trend with partner risk being one of the most significant contributors to risk perceptions [4, 16, 27]. However, condom use is observed to be lower in these aging populations of women [3, 16]. This is critical for intervention development, as research indicates that minority women perceive their HIV risk to be lower than actual risk [28].

Consistent with other studies [3, 16, 25, 29], our findings showed that religion and spirituality were central to descriptions of Dominican cultural beliefs and practices. Some participants expressed believing their spouses to be faithful because monogamy is a part of their religious beliefs. Catholicism, specifically does not support condom use as sex is believed to be only for procreation, further contributing to high risk sexual practices and difficulties with condom negotiation [3].

Marianismo, encouraging sexual passivity in women and machismo encouraging relationship dominance, sexual risk taking behaviors and multiple sexual partnerships in men, are commonly known aspects of Latino culture and important themes that emerged [3, 16, 25]. Such standards, coupled with religious beliefs, further increases women’s vulnerability to HIV [3, 16]. Additionally, HIV-related stigma is also proven to interfere with prevention efforts and ultimately behavior change. HIV-related stigma prevents HIV testing and health information seeking behaviors in populations of women midlife and older, evidenced in the story told by a participant about a widow being insulted when asked to take an HIV test [5, 29]. The potential of women being stigmatized for initiating discussions about safe sex practices within their intimate partnerships, also perpetuates high risk behaviors, particularly in primary partnerships [12, 29]. Although studies have shown a decrease in perceptions of HIV risk for women who are married and in steady partnerships [29], most of our focus group participants did not. They identified the travel of their husbands and steady partners back and forth to the Dominican Republic as an ongoing concern about fidelity and HIV risk. Perceptions of health threats is determined by a sense of susceptibility, which results in behavioral responses influenced by preventive actions [27]. With an estimated 67,000 in the Dominican Republic living with HIV/AIDS and the second highest HIV incidence rates in the Caribbean, there is tremendous cause for concern amongst Dominican women midlife and older [https://www.aidshealth.org/global/dominican-republic/].

Other life experiences associated with the aging process also increases the vulnerability of older women to HIV [30]. Widowhood, loss of meaningful spousal roles, loss of friends, and diminished economic resources can contribute to a potential decline in self-esteem and interpersonal power [30, 31]. Moreover, vulnerable older women will find their pool of available partners diminished [31, 32]. These aging-related reasons can result in difficulties negotiating condom use and in implementing other safe sex practices [31, 32]. In younger women, socioeconomics alone was the greatest contributor to decreased self-efficacy for condom negotiation [33]. A study of older women, many ethnically diverse, 38% reported high risk sexual behavior for fear of loneliness [34]. Research also indicates that changes in physical health status with age are also associated with negative self-perceptions and feelings of powerlessness; mentioned often in our focus group discussions around partner fidelity [5, 34]. These aging-related reasons can result in feelings of social devaluation, making them open to non-monogamous intimate partnerships [5, 34].

**Personal risk reasons (Cathexis)** identified by our participants include knowledge gaps that may diminish perceptions of HIV risk, persistent in populations of older women. In a study of Black women, midlife and older, only 18% of participants knew condoms were effective in preventing the transmission of HIV [31]. Our focus group participants acknowledged generational differences in perception of HIV risk. While campaigns targeting older adults with knowledge about HIV transmission risk have emerged, risk reduction materials remain sparse [27]. Further increasing women’s vulnerability is the lack of discussion around sexual and drug use behaviors with primary care providers [8, 27]. Although not asked directly in focus group discussions, providers were never mentioned as a source of prevention information. These results potentially suggest, as has been shown by other studies, that there is a need for primary care providers to deliver culturally-specific HIV prevention information for their aging populations [27]. Providers are also recommended to address self-esteem issues in aging populations of women of color, including the societal and cultural reasons around aging that may increase their susceptibility to HIV [3]. Studies have also found provider endorsement of HIV testing amongst Latino women, to be significant [3]. This indicates the critical need for providers to have conversations around sexual health with patients, as the likelihood of testing decreases with increasing age [3, 7]. Yet, it remains uncomfortable for many providers to address sexual health during visits, with only 9 % of both men and women reporting such conversations during routine medical care [35]. Older adults have greater risk reasons for HIV transmission [35]. However, as a result of HIV risk prevention efforts being traditionally focused on youth, [16, 36] the ongoing beliefs that sexuality should not be a concern of the mature does not prepare patients nor their providers for the necessary and critical conversations [37]. Specific to women midlife and older, partner risk-related communication should support the recognition of multiple sexual partnerships with both men and women, and signs and symptoms of substance and drug abuse, for effective risk reduction [3, 16, 28]. In fact, preventive measures and testing was a major focus group theme, indicating the need for improved self-efficacy. Although not identified as a theme, drug abuse as it relates to HIV risk was discussed several times during the focus group discussions. Additional research on the sexuality of the mature, should focus on the reasons that both mediate and moderate patient and provider communication around sexual health [36, 37].

### Limitations

There are several potential limitations associated with this study. These include the lack of multiple measures to assess study constructs, such as data triangulation and a constant comparison method to assess findings derived from two or more sources. A convenience sample was used to collect data that may produce effects regarding self-selection, lack of generalizability and socially desirable bias. Focus group members may be hesitant to express thoughts or dominant voices may override others.

## Conclusion

Our study explored the perceptions of HIV risk, and identified themes that describe the socio-cultural and contextual reasons that contribute to perceptions of risk.in a group of HIV concerned Dominican women 50 years of age and over that could have implications for HIV prevention interventions. Based on our finding, women 50 and over could benefit greatly from interpersonal skills training to support educational limitations, literacy barriers and the lack of occupational skills. Interpersonal skill building can improve self-efficacy for greater control over their livelihood and will be addressed in future research activities. Older women with inadequate literacy will need specialized interventions to ensure the understanding of health education messages, and the effective comprehension of sexual risk. Social capital, defined as descriptions of social structures that facilitate collective action, are proven to be a strong predictor of HIV infection rates. An understanding of such salient reasons that impact risk behaviors among older Latino women is essential for policy updates and development; evidenced in the recent CDC call for the identification of country-level differences in HIV risk reasons for Hispanic populations. As research shows the decrease likelihood of older adults to adopt prevention strategies, our study supports intervention research for targeted risk reduction in vulnerable aging populations.

## Data Availability

Data are available upon reasonable request from the corresponding author.

## Abbreviations

CDC: Centers for Disease Control and Prevention
HIV/AIDS: Human Immunodeficiency Virus/ Acquired Immunodeficiency Syndrome
PLWH: People Living with HIV/AIDS
STI: Sexually Transmitted Infection
TGP: Theory of Gender and Power
